# Standardized treatment and determinants on 9,059 syphilis-infected pregnant women during 2015–2018 in Hunan, China

**DOI:** 10.1038/s41598-020-69070-3

**Published:** 2020-07-21

**Authors:** Huixia Li, Jingjing Tan, Zhongwen Luo, Jianfei Zheng, Guangwen Huang, Juan Xiao, Qun Huang, Na Feng

**Affiliations:** 1 Department of Child Health Care, Hunan Provincial Maternal and Child Health Care Hospital, Changsha, 410008 Hunan Province China; 2 NHC Key Laboratory of Birth Defect for Research and Prevention, Hunan Provincial Maternal and Child Health Care Hospital, Changsha, 410008 Hunan Province China; 3 Department of Emergency Medicine, Second Xiangya Hospital, Central South University, Changsha, 410011 Hunan Province China; 4 Department of Health Care, Shenzhen Nanshan Maternal and Child Health Care Hospital, Shenzhen, 518067 Guangdong Province China

**Keywords:** Infectious diseases, Epidemiology

## Abstract

This study was aimed to describe the standardized treatment rate of syphilis-infected pregnant women in Hunan province and to explore the determinants for standardized treatment. All syphilis-infected pregnant women registered in the Information System of Prevention of Mother-to-Child Transmission of Syphilis Management (IPMTCT) in Hunan between January 2015 and December 2018 were included in this study. Among 9,059 pregnant women with syphilis, 7,797 received syphilis treatment, with a treatment rate of 86.1%, and 4,963 underwent standardized syphilis treatment, with an average standardized treatment rate of 54.8%. The facilitators for the standardized treatment included abnormal reproductive histories (aOR = 1.15, 95%CI:1.03–1.28), time of first prenatal care within 1–12 weeks (aOR = 5.17, 95%CI:4.19–6.37) or within 13–27 weeks (aOR = 5.56, 95%CI:4.46–6.92), previous syphilis infection (aOR = 1.64, 95%CI: 1.48–1.81), and definite syphilis infection status of sexual partner (negative: aOR = 1.73, 95%CI:1.57–1.91; positive: aOR = 1.62, 95%CI:1.34–1.95). The barriers included marital status being unmarried/divorced/widowed (aOR = 0.81, 95%CI: 0.65–0.99), pluripara (aOR = 0.58, 95%CI: 0.46–0.74), number of children ≥ 2 (aOR = 0.45, 95%CI: 0.35–0.57), and syphilis clinical stage being primary/secondary/tertiary (aOR = 0.72, 95%CI: 0.58–0.88) or unclear (aOR = 0.78, 95%CI: 0.70–0.86). Though the treatment rate of syphilis-infected pregnant women was high, the standardized treatment rate was low. The facilitators and barriers on standardized treatment of gestational syphilis were identified at the patient level.

## Introduction

Syphilis is a chronic sexually-transmitted disease caused by *Treponema pallidum* infection and invades multiple systems. The epidemic situation of syphilis is severe in China. Data from National Bureau of Statistics of China show the reported case number of syphilis in 2017 was 475,860 and the reported incidence was 34.5/100,000, which ranked first among all sexually-transmitted diseases. Gestational syphilis, the syphilis occurring or discovered during pregnancy, includes progestational or gestational syphilis and is dominated by latent infection. *T. pallidum* can be transmitted via the placenta to the fetus. The incidence rates of gestational syphilis in China are 0.2–0.5%^1–3^, which are lower than the levels in Africa (1.1%) and South America (1.6%)^4,5^. The untreated gestational syphilis can cause severe adverse pregnancy outcomes, such as spontaneous abortion, stillbirth, premature delivery, low birth weight, neonatal death and congenital syphilis, and the incidence rates of overall adverse pregnancy outcomes are up to 25–77%^6–9^.

To eliminate the mother-to-child transmission of syphilis and improve the pregnancy outcomes of syphilis-infected pregnant women, China integrated the "screening, diagnosis, treatment and detected follow-up" comprehensive intervention measures into the *Prevention of Mother-to-Child Transmission of Syphilis* (PMTCT) project and applied to the whole country in 2011. The key measure against the mother-to-child transmission of syphilis is to use prompt, full-course and sufficient penicillin treatment. The PMTCT implementation plan in 2011 recommended the therapeutic drug of benzathine penicillin or procaine penicillin, the replacement with ceftriaxone in the unavailability of penicillin, and the use of erythromycin for penicillin-allergic patients. Along with the implementation of PMTCT in China, the prenatal syphilis detection rate and treatment rate among pregnant women were significantly improved from 67.4 to 94.1% and from 58.8 to 89.4% respectively from 2012 to 2016^10^. However, the standardized treatment rates of syphilis were only less than 60%^6,10,11^, which severely reduced the mother-to-child transmission blocking effect. Hunan province initiated PMTCT among 21 counties in 2011, then extended to 48 counties in 2014 and covered all 123 counties in 2015. During the initial five years of the implementation of PMTCT in Hunan, the prenatal syphilis detection rate among pregnant women was significantly increased from 44.2% in 2011 to 90.8% in 2015, but the maternal syphilis treatment rate, especially the standardized treatment rate, was still unclear^12^.

The existing research on gestational syphilis treatment is focused on congenital syphilis and other adverse pregnancy outcomes^13–16^, but rarely on the situation or determinants on standardized treatment of gestational syphilis^17^. Studying the determinants on standardized treatment of gestational syphilis helps to identify the barriers and facilitators for standardized treatment and to underlie the further improvement of standardized treatment rates, thereby promoting the effective implementation of measures against mother-to-child syphilis transmission. In this study, the surveillance data about PMTCT of syphilis implemented in Hunan province of China between 2015 and 2018 were used. Then the determinants on standardized treatment of syphilis among pregnant women were investigated, which scientifically underlay the management of gestational syphilis.

## Materials and methods

### Subjects

The Information System of Prevention of Mother-to-Child Transmission of Syphilis (IPMTCT) is a nationwide health facilities-based case report system established in 2011 and has been used to monitor and evaluate the epidemics of maternal syphilis and congenital syphilis in China. All syphilis-infected pregnant women registered in the IPMTCT of Hunan between January 2015 and December 2018 were included in this study. The inclusion criteria were: (1) pregnant women at age ≥ 18; (2) syphilis-infected pregnant women who were positive in both non-*T. pallidum* antigen serological (NTPAS) tests and *T. pallidum* antigen serological (TPAS) tests; (3) diagnosis time of syphilis infection being between January 1, 2015 and December 31, 2018. The exclusion criteria were: (1) non-resident pregnant women; (2) penicillin-allergic patients.

### Syphilis infection and diagnostic criteria

Health facilities provided all pregnant women with free syphilis screening tests in the first prenatal care by using NTPAS test, which were either rapid plasma reagin ring card (RPR) test or toluidine red unheated serum (TRUST) test. The patients with positive results in the NTPAS test were confirmed using TPAS tests, including *T. pallidum* particle assay (TPPA), *T. pallidum* haemagglutination assay (TPHA) or enzyme-linked immunosorbent assay (ELISA). According to *Prevention of Mother-to-Child Transmission of HIV/AIDS, Syphilis and Hepatitis B*, syphilis infection was diagnosed as being positive in both NTPAS tests (RPR or TRUST) and TPAS tests (TPPA or TPHA or ELISA). The syphilis was classified by *Gestational Syphilis and Congenital Syphilis Prevention and Treatment Guideline* into the latent, primary, secondary and tertiary stages^18^.

### Treatment of syphilis

Pregnant women were immediately treated upon the discovery of syphilis infection. The first choice for syphilis treatment was penicillin, which was recommended to be either benzathine penicillin or procaine penicillin G. For unavailablity of penicillin or for penicillin allergy, the optional replacing antibiotic was either ceftriaxone or erythromycin. The syphilis-infected pregnant women discovered at the first trimester of pregnancy received 1 course of treatment at both the first and third trimesters. The syphilis-infected pregnant women discovered at the second or third trimester were immediately given a 2-course treatment, and the interval between the two courses was above 4 weeks (at least 2 weeks), and the second course was started at the third trimester and should be finished 1 month before labor. Those who were diagnosed right before labor were given 1 course of treatment. During the treatment, patients with reoccurrence or secondary infection were given another course of treatment.

A standardized treatment was defined as a 2-course treatment with sufficient penicillin during pregnancy (e.g. "1 course" for benzathine penicillin was defined as 1 time each week for three consecutive times; "1 course" for procaine penicillin G was defined as 1 time each day for 15 consecutive days), the interval between the 2 courses was above 2 weeks, and the 2nd course was conducted and finished at the third trimester. The non-standardized treatment referred to no reception of full-course and sufficient penicillin treatment, or reception of non-penicillin replaced treatment during the pregnancy, or conduction of syphilis treatment only 1 month before labor.

### Data collection

The registration data of syphilis-infected pregnant women from the Hunan IPMTCT included social demographic data, reproductive histories, syphilis infection and treatment. Specifically, social demographic data included age, ethnicity, residence, education level, occupation, and marital status. Information about reproductive histories included gravidity, parity, abnormal reproductive histories, pregnancy complications, and number of children. Information about syphilis diagnosis and treatment included time of first prenatal care, period of syphilis screening and diagnosis, syphilis clinical stage, previous syphilis infection, syphilis treatment, and syphilis infection status of sexual partner.

### Definition of variables

The age of pregnant women was divided into 5 groups: < 20, 20–24, 25–29, 30–34 and ≥ 35. Ethnicity included Han and minorities. Residence was separated into nonurban and urban. The education level was classified into primary school or below, middle school, and college or above. Occupation was divided into farmers, housework or unemployment, and others. The marital status was divided into married, and unmarried/divorced/widowed. The time of first prenatal care was separated into the first trimester (1–12 weeks), second trimester (13–27 weeks) and third trimester (≥ 28 weeks). The period of syphilis diagnosis was divided into the first trimester (1–12 weeks), second trimester (13–27 weeks), third trimester (≥ 28 weeks) and intrapartum/postpartum. The clinical stage of syphilis was divided into the latent, primary, secondary and tertiary stages. Previous syphilis infection meant that a pregnant woman had been confirmed with positive syphilis infection before the last menstrual period of this pregnancy, and was obtained based on her self-report for the syphilis infection status. The syphilis infection status of the sexual partner was considered as unclear (unexamined/unknown), negative and positive.

### Statistical analysis

Data were analyzed on SPSS 25.0 (IBM, Chicago, USA). The standardized treatment rates of syphilis-infected pregnant women with different characteristics were compared using chi-square test. With "whether or not the pregnant women received a standardized treatment during pregnancy" as the dependent variable (yes = 1, no = 0), the significant variables identified by univariate analysis were sent into a multivariate logistic regression model, and the adjusted odds ratio (aOR) and 95% confidence interval (CI) of each variables were calculated. The selection of independent variables was based on forward stepwise regression (α_in_ = 0.05, α_out_ = 0.10). The statistical significance was set at *P* < 0.05.

### Ethics approval

All syphilis-infected pregnant women were required to complete a special questionnaire developed by the national PMTCT program to register the information about their syphilis screening, diagnosis and treatment. Prenatal syphilis screening, diagnosis and treatment were conducted following the guidelines and regulations of the integrated National PMTCT Program of HIV, Syphilis and Hepatitis B. As syphilis-infected pregnant women had signed informed consent in the PMTCT program, a special informed consent for this retrospective study was not required. Given these reasons, the Ethics Committee of Hunan Provincial Maternal and Child Health Care Hospital approved our study and waived the informed consent. The retrospective study was conducted in accordance with the Declaration of Helsinki. Strict confidentiality was adhered to regarding any data collected throughout the study.

## Results

### Treatment status of pregnant women with syphilis

Initially, 9,289 pregnant women with syphilis were enrolled. After 137 non-resident cases, 39 cases aged < 18 years and 54 penicillin-allergic cases were excluded, a total of 9,059 cases were finally involved in our analyses. Of them, 7,797 cases received syphilis treatment, with a treatment rate of 86.1% (7,797/9,059), and 4,963 cases underwent the standardized syphilis treatment, with an average standardized treatment rate of 54.8% (4,963/9,059). The standardized treatment rate increased from 29.9% (488/1631) in 2015 to 72.9% (2,123/2,912) in 2018. Moreover, 4,096 subjects received non-standardized treatment (including 1,262 cases of non-treatment during pregnancy; 178 cases of non-penicillin replaced treatment; 2,656 cases with insufficient penicillin treatment), with a non-standardized treatment rate of 45.2% (4,096/9,059). The treatment regimen was dominated by benzathine penicillin (84.1%, Table 1).

**Table 1:** Treatment for pregnant women with syphilis.

Syphilis treatment	Frequency	Rate, %
**Treatment**		
Yes	7,797	86.1
No	1,262	13.9
**Standardized treatment**		
Yes	4,963	54.8
No	4,096	45.2
Non-treatment	1,262	13.9
Non-penicillin treatment	178	2.0
Insufficient course for penicillin treatment	2,656	29.3
**Standardized treatment rate from 2015–2018**		
2015	488/1631	29.9
2016	914/1983	46.1
2017	1,438/2,533	56.8
2018	2,123/2,912	72.9
**Treatment regimens**		
Benzathine penicillin	7,619	84.1
Procaine penicillin G	92	1.1
Ceftriaxone	86	0.9
Non-treatment	1,262	13.9
Total	9,059	100.0

### Characteristics of pregnant women with syphilis

The 9,059 pregnant women with syphilis were aged 29.4 ± 5.6 years, ranging from 18 to 50 years, and mostly (34.3%) were aged 25–29 years. The ethnicity was dominated by Han (87.2%). About 51.3% of them lived in nonurban areas. The education level was mostly middle school (86.3%), and the occupation was mostly "housework or unemployment" (48.1%). About 94.6% of them were married, and 68.3% of them were pluriparas. About 66.0% of them underwent the first prenatal care at ≤ 12 weeks; 68.9% of them were diagnosed at < 28 weeks, and 67.2% of them suffered latent syphilis. Moreover, 70.0% of them had no previous syphilis infection, and 62.1% had sexual partners with unclear syphilis infection (Table 2).

**Table 2:** Characteristics and standardized treatment rate for pregnant women with syphilis.

Characteristics	Frequency (n = 9,059)	Proportion, %	Standardized treatment			
			Number	Rate, %	$\chi^{2}$	*P* value
Maternal age (years)					33.860	0.000*
< 20	213	2.4	106	49.8		
20–24	1573	17.4	831	52.8		
25–29	3,105	34.3	1815	58.5		
30–34	2,398	26.5	1,315	54.8		
≥ 35	1,770	19.5	896	50.6		
Ethnicity					24.553	0.000*
Han	7,903	87.2	4,408	55.8		
Minorities	1,156	12.8	555	48.0		
Residence					0.433	0.511
Nonurban	4,648	51.3	2,562	55.1		
Urban	4,411	48.7	2,401	54.4		
Education level					17.638	0.000*
Primary school or below	676	7.5	324	47.9		
Middle school	7,816	86.3	4,303	55.1		
College or above	567	6.3	336	59.3		
Occupation					100.822	0.000*
Farmer	2,932	32.4	1,413	48.2		
Housework or unemployment	4,360	48.1	2,614	60.0		
Others	1767	19.5	936	53.0		
Marital status					10.944	0.001*
Married	8,569	94.6	4,730	55.2		
Unmarried/divorced/widowed	490	5.4	233	47.6		
Gravidity					13.087	0.004*
Primigravidae	1757	19.4	1,024	58.3		
1 previous pregnancy	2,892	31.9	1,590	55.0		
2 previous pregnancy	2,130	23.5	1,143	53.7		
≥ 3 previous pregnancy	2,280	25.2	1,206	52.9		
Parity					173.436	0.000*
Primipara	2,869	31.7	1862	64.9		
Pluripara	6,190	68.3	3,101	50.1		
Abnormal reproductive histories					9.306	0.002*
Yes	1972	21.8	1,140	57.8		
No	7,087	78.2	3,823	53.9		
Pregnancy complications					0.429	0.513
Yes	1,037	11.4	578	55.7		
No	8,022	88.6	4,385	54.7		
Number of children					404.889	0.000*
0	3,184	35.1	2025	63.6		
1	4,282	47.3	2,410	56.3		
≥ 2	1593	17.6	528	33.1		
Time of first prenatal care					457.219	0.000*
1–12 weeks of gestation	5,978	66.0	3,457	57.8		
13–27 weeks of gestation	2,360	26.1	1,385	58.7		
≥ 28 weeks of gestation	721	8.0	121	16.8		
Period of syphilis diagnosis					3,919.722	0.000*
1–12 weeks of gestation	4,236	46.8	3,185	75.2		
13–27 weeks of gestation	1998	22.1	1534	76.8		
≥ 28 weeks of gestation	507	5.6	244	48.1		
Intrapartum/postpartum	2,318	25.6	0	0.0		
Clinical stage of syphilis					94.404	0.000*
Latent syphilis	6,092	67.2	3,553	58.3		
Primary/secondary/tertiary syphilis	447	4.9	219	49.0		
Unclear	2,520	27.8	1,191	47.3		
Previous syphilis infection					157.580	0.000*
Yes	2,717	30.0	1761	64.8		
No	6,342	70.0	3,202	50.5		
Syphilis infection status of sexual partner					209.712	0.000*
Unclear	5,626	62.1	2,752	48.9		
Negative	2,856	31.5	1859	65.1		
Positive	577	6.4	352	61.0		
Total	9,059	–	4,963	54.8	–	–

*P < 0.05.

### Univariate analysis for standardized treatment of syphilis-infected pregnant women

Univariate analysis showed that the factors related with standardized treatment of syphilis-infected pregnant women included the maternal age, ethnicity, education level, occupation, marital status, gravidity, parity, abnormal reproductive histories, number of children, time of first prenatal care, period of syphilis diagnosis, syphilis clinical stage, previous syphilis infection, and syphilis infection status of sexual partner (Table 2). The unrelated factors were residence and pregnancy complications.

### Multivariate analysis for standardized treatment of syphilis-infected pregnant women

Multivariate logistic regression analysis showed the facilitators for the standardized syphilis treatment in pregnant women included abnormal reproductive histories (aOR = 1.15, 95%CI: 1.03–1.28), time of first prenatal care within 1–12 weeks (aOR = 5.17, 95%CI: 4.19–6.37) or 13–27 weeks (aOR = 5.56, 95%CI: 4.46–6.92), previous syphilis infection (aOR = 1.64, 95%CI: 1.48–1.81), and definite syphilis infection status of sexual partner (negative: aOR = 1.73, 95%CI: 1.57–1.91; positive: aOR = 1.62, 95%CI: 1.34–1.95). The barriers included marital status being unmarried/divorced/widowed (aOR = 0.81, 95%CI: 0.65–0.99), pluripara (aOR = 0.58, 95%CI: 0.46–0.74), number of children ≥ 2 (aOR = 0.45, 95%CI: 0.35–0.57), clinical stage of syphilis being primary/secondary/tertiary (aOR = 0.72, 95%CI: 0.58–0.88) or unclear (aOR = 0.78, 95%CI: 0.70–0.86) (Table 3).

**Table 3:** Multivariate logistic regression of standardized treatment for pregnancy women with syphilis (standardized treatment = 1, non-standardized treatment = 0).

Characteristics	*β*	SE	Wald $\chi^{2}$	*P* value	*aOR*	95%*CI*
Marital status						
Married					1.00	
Unmarried/divorced/widowed	– 0.21	0.11	3.96	0.047	0.81*	0.65–0.99
Parity						
Primipara					1.00	
Pluripara	– 0.54	0.12	20.35	0.000	0.58*	0.46–0.74
Abnormal reproductive histories						
Yes	0.14	0.06	6.05	0.014	1.15*	1.03–1.28
No					1.00	
Number of children			194.77	0.000		
0					1.00	
1	0.10	0.12	0.72	0.400	1.11	0.88–1.39
≥ 2	– 0.81	0.13	40.06	0.000	0.45*	0.35–0.57
Time of first prenatal care			250.29	0.000		
1–12 weeks of gestation	1.64	0.11	237.15	0.000	5.17*	4.19–6.37
13–27 weeks of gestation	1.72	0.11	235.51	0.000	5.56*	4.46–6.92
≥ 28 weeks of gestation					1.00	
Clinical stage of syphilis			30.14	0.000		
Latent syphilis					1.00	
Primary/secondary/tertiary syphilis	– 0.33	0.11	10.18	0.001	0.72*	0.58–0.88
Unclear	– 0.25	0.05	23.88	0.000	0.78*	0.70–0.86
Previous syphilis infection						
Yes	0.49	0.05	92.29	0.000	1.64*	1.48–1.81
No					1.00	
Syphilis infection status of sexual partner			127.37	0.000		
Unclear					1.00	
Negative	0.55	0.05	117.35	0.000	1.73*	1.57–1.91
Positive	0.48	0.10	25.19	0.000	1.62*	1.34–1.95

AOR denotes adjusted odds ratio. CI denotes confidence interval. *P < 0.05.

## Discussion

The first-choice antibiotic for syphilis-infected pregnant women was penicillin, and the basic treatment principles were early discovery, and early, sufficient and full-course treatment. The syphilis treatment at the first trimester prevented the embryo from infection, and the treatment at the third trimester cured both the pregnant woman and the infected embryo, so the infected embryo can be cured before labor. Thus, the standardized treatment of gestational syphilis required for sufficient penicillin treatment for 2 courses during the pregnancy and the 2nd course should be conducted and finished at the third trimester. Since the extensive implementation of PMTCT in 2011, except for some developed cities such as Shenzhen^19^, the standardized treatment rates of gestational syphilis are unfavorable in other cities and are only 44.6–65.1%^2,6,20,21^. There is still huge gap from the goal of eliminating mother-to-infant syphilis transmission by 95% set up by Worth Health Organization in 2014^22^. After the implementation of national PMTCT in China for 8 years, many studies have been made to report the implementation effects of this project, including the process indexes (prenatal syphilis screening rates, gestational syphilis treatment rates) and effect indexes (congenital syphilis occurrence)^2,3,10,20^. However, there is no report about the determinants on standardized treatment of gestational syphilis. Results show the facilitators for the standardized syphilis treatment include abnormal reproductive histories, time of first prenatal care < 28 weeks, previous syphilis infection, and definite syphilis infection status of sexual partner (negative or positive). The barriers include marital status being unmarried/divorced/widowed, pluripara, number of child ≥ 2, and clinical stage of syphilis being primary/secondary/tertiary or unclear. A better understanding of syphilis standardized treatment as well as the associated facilitators and barriers will help health care providers to identify high-risk populations and improve syphilis standardized treatment. Moreover, the results can provide scientific evidence for formulating and optimizing intervention strategies against mother-to-child transmission of syphilis.

Due to the implementation of the PMTCT in Hunan, the standardized treatment rate among pregnant women with syphilis was significantly improved from 29.9% in 2015 to 72.9% in 2018. Such improvement is attributed to the huge investment and policy support from the national and provincial governments on PMTCT in the past eight years, including laboratory construction of health facilities, special technical training about intervention measures of PMTCT for health care providers, and free syphilis detection and treatment for all pregnant women^23^. Although the standardized treatment rate of syphilis-infected pregnant women in Hunan has improved in recent years, the average standardized treatment from 2015 to 2018 is 54.8%, which is similar to that in Zhejiang (54.7%)^2^ and lower than that in Shenzhen (89.8%)^19^. Shenzhen is one of the most developed cities of China and is severely attacked by syphilis breakout. To prevent and control congenital syphilis, Shenzhen experimentally implemented PMTCT in the whole city as early as 2002. After 10 years of comprehensive intervention measures and practice, Shenzhen made significant success in PMTCT, as the incidences of congenital syphilis dropped from 115/100,000 live births in 2002 to 10/100,000 live births in 2011^13^. Along with the successful experimental trial in Shenzhen, PMTCT was expanded in 2011 to 1,156 counties in China. Hunan initiated PMTCT as late as 2011 in 21 counties, then extended to 48 counties in 2014 and covered all 123 counties in 2015. Hunan carried out PMTCT 10 years later than Shenzhen and thus lacked experience in organization, management and inter-department cooperation of PMTCT, which moderately hindered the implementation of mother-to-child transmission blocking measures.

During the implementation of PMTCT in China, the health facilities initiatively offered free syphilis serological tests for pregnant women during their first prenatal care. Thus, we find the time of first prenatal care is closely and collinearly correlated with the time of syphilis diagnosis, which explains why only the time of first prenatal care was included into the multivariate logistic regression model. The time of gestational weeks in the first prenatal care was 13.5 weeks on average and was < 28 weeks in 92.1% of the patients. The diagnosis time of gestational syphilis was 19.0 weeks on average and was < 28 weeks in 68.9% of the patients, but was intrapartum or postpartum in 25.6% of the patients. Though the prenatal care rates in the first and second trimesters were high, the syphilis diagnosis rates in these two trimesters were lower, which was mostly because the pregnant women deliberately hid the disease and rejected syphilis detection during the prenatal care. This study shows that the time of first prenatal care is a key factor deciding the standardized treatment of syphilis-infected pregnant women, and the probabilities of receiving syphilis standardized treatment during the first prenatal care in the first and second trimesters are 5.17 and 5.56 times of those in the third trimester, respectively. Similarly, qualitative studies in Congo and Zambia demonstrated that the belated time of first prenatal care was a major factor that hindered the detection and treatment of syphilis in pregnant women^17^. A retrospective study in South Africa showed that the compliance with benzathine penicillin treatment for syphilis during pregnancy was significantly associated with the time of first prenatal care visit, and that women without treatment presented 3–4 weeks later on average for their first visit than those women receiving complete treatment^24^. The standardized treatment of gestational syphilis should be finished by using 2 courses of sufficient penicillin, and the two courses should both last 15 days or 3 weeks, and be separated by at least 2 weeks. Thus, those who received syphilis screening only in the third trimester, especially intrapartum, cannot complete 2 courses of syphilis treatment. Health care providers should strengthen social propaganda and health education on mother-to-child transmission of syphilis, increase the syphilis screening rates of pregnant women in the first and second trimesters, and diagnose syphilis infection as soon as possible to further improve the standardized treatment rate of gestational syphilis.

Gestational syphilis management also requires for syphilis detection over the sexual partners of syphilis-infected pregnant women. If syphilis infection of sexual partners is confirmed, a prompt and standardized treatment is needed to prevent the pregnant women from repeated infection. In this study, the syphilis infection status is unclear in up to 62.1% of the sexual partners, indicating the notification and syphilis detection rates among the sexual partners of syphilic pregnant women are low. Fear of domestic violence among pregnant women, poor communication between partners, and syphilis-related stigma were the possible barriers for their partner notification and detection for syphilis^25^. The pregnant women whose sexual partners had definite syphilis infection status may have strong consciousness of prenatal care and can fully understand the importance of notifying the sexual partners, and get enough family support, so their standardized treatment rates are high. The syphilis-infected pregnant women with abnormal reproductive histories feared of infecting their embryos and of having adverse pregnancy outcomes again, so their standardized treatment rates were high. On the contrary, multiparas, or women already having ≥ 2 children may have already had healthy children, and they were not very desiring to protect the fetuses from infection, which led to the very low rate of standardized treatment among them. The pregnant women with syphilis being unmarried/ divorced/widowed may lack social and family support and may have stigma, so the standardized treatment rate was low among them.

In this study, 30.0% of the pregnant women with syphilis had a previous syphilis infection, and 70.0% of them were infected for the first time. The knowledge rates of syphilis-caused health hazards, detection and treatment, and the detection and treatment compliance rates are high among those with a previous syphilis infection, which account for the high standardized treatment rate among them. Gestational syphilis is dominated by latent infection, without showing any clinical symptom, and can be identified by routine prenatal serological tests^18^. Our study shows the standardized treatment rates differ among patients at different clinical stages of syphilis. The main symptom of primary syphilis is small and painless lesions, called chancres, in areas of sexual contact. Because chancres are painless and disappear spontaneously, many pregnant women do not seek treatment. Moreover, the secondary syphilis and tertiary syphilis inherently result from the untreatment or non-standardized treatment of primary syphilis or early syphilis^26^.

This study has some limitations. First, the data were obtained from IPMTCT in Hunan, which is a passive surveillance system. Since data collection on maternal syphilis is completely dependent on health facility reporting, our study may be biased by a high proportion of unclear data (missing data) on some important factors, such as clinical stage of syphilis and syphilis infection status of sexual partner. Second, syphilis infection was diagnosed as being positive in both NTPAS tests and TPAS tests. However, a few pregnant women with previous syphilis infection may have syphilis serum-resistance, their serum titers of NTPAS tests are maintained at low levels (≤ 1:8) for a long time (even for life)^18^, which will be lead to a diagnosis bias for current syphilis infection. Third, due to data limitation, the determinants of gestational syphilis standardized treatment considered here only include patient-level factors, but exclude the factors at the health system level or health care provider level. Two qualitative studies in Africa found that the key barriers against implementation of syphilis testing and treatment were fragmentation of the health system and low accessibility of health care service (geographical and functional) in the health system level, and lack of knowledge and training about evolving best syphilis practices in health care providers^17,27^. Thus, some key factors related to gestational syphilis standardized treatment may have not been found, which largely restrains the improvement of gestational syphilis standardized treatment rates in Hunan. Fourth, the *2015 National PMTCT Guidelines* specifically requested that during the gestational syphilis therapeutic process, if the NTPAS test showed titer rise or the result changed from "negative" to "positive", it was judged as reoccurrence or reinfection and 1 new course of treatment should be supplemented^23^. Since the patients with reoccurrence or reinfection totally need 3 courses of penicillin treatment, the requirements to their standardized treatment are different from regular pregnant women with syphilis. However, since the reoccurrence or reinfection of gestational syphilis was not excluded in our study, the rate of standardized treatment among pregnant women with syphilis may be overestimated.

## Conclusions

Though the treatment rate of syphilis-infected pregnant women was high, the syphilis standardized treatment rate was low. The facilitators and barriers on standardized treatment of gestational syphilis were identified at the patient level. Further studies should be expanded to the determinants on standardized treatment of gestational syphilis at the health system level and the health care provider level.

## Data Availability

The datasets generated during and/or analysed during the current study are not publicly available due to the personal privacy of subjects but are available from the corresponding author on reasonable request.
